# Review of *Parathoracaphis* Takahashi, 1958 with description of a new species from China (Hemiptera, Aphididae, Hormaphidinae)

**DOI:** 10.3897/zookeys.623.10205

**Published:** 2016-10-11

**Authors:** Jing Chen, Li-Yun Jiang, Ge-Xia Qiao

**Affiliations:** 1 Key Laboratory of Zoological Systematics and Evolution, Institute of Zoology, Chinese Academy of Sciences, No. 1 Beichen West Road, Chaoyang District, Beijing 100101, P.R. China

**Keywords:** aphid, key, morphology, new taxa, Nipponaphidini

## Abstract

The aphid genus *Parathoracaphis* Takahashi, 1958 is reviewed. *Parathoracaphis spinapilosa* **sp. n.**, found on *Quercus* sp. and on an unidentified species of Fagaceae in China is described and illustrated. A generic diagnosis and a key to *Parathoracaphis* species are presented.

## Introduction

The aphid genus *Parathoracaphis* was erected by Takahashi (1958), with *Thoracaphis setigera* Takahashi, 1932 as the type species, based on the morphological characters that head and thorax are completely fused with abdominal segments I–VII, dorsum lacks pustules but bears spine-like submarginal setae, and siphunculi absent. *Thoracaphis elongata* Takahashi, 1941 and *Thoracaphis kayashimai* Takahashi, 1950 were also assigned to this genus (Takahashi 1958). Eastop and Hille Ris Lambers (1976) subsequently included two other species, *Thoracaphis cheni* Takahashi, 1936 and *Thoracaphis gooti* Takahashi, 1950, under *Parathoracaphis*. Ghosh (1988) considered *Hoplothoracaphis* Pramanick, Samanta & Raychaudhuri, 1983 as a synonym of *Parathoracaphis*, and consequently referred *Hoplothoracaphis manipurensis* Pramanick, Samanta & Raychaudhuri, 1983 to *Parathoracaphis*.

Herein, a new species *Parathoracaphis spinapilosa* sp. n. is described, found on *Quercus* sp. and an unidentified species of Fagaceae in Fujian and Yunnan, China. Therefore, the genus *Parathoracaphis* now includes seven species: *Parathoracaphis cheni* (Takahashi), *Parathoracaphis elongata* (Takahashi), *Parathoracaphis gooti* (Takahashi), *Parathoracaphis kayashimai* (Takahashi), *Parathoracaphis manipurensis* (Pramanick, Samanta & Raychaudhuri), *Parathoracaphis setigera* (Takahashi), and *Parathoracaphis spinapilosa* sp. n.

## Materials and methods

*Morphological description*. Aphid terminology in this paper generally follows Takahashi (1958) and Ghosh (1988). The unit of measurements is millimetres (mm). In Table 1, the following abbreviations are used: Ant.IIIBD, basal diameter of antennal segment III; URS, ultimate rostral segment; BW URS, basal width of ultimate rostral segment; 2HT, second hind tarsal segment; BW Cauda, basal width of cauda.

**Table 1.** Morphometric data of *Parathoracaphis spinapilosa* sp. n.

Parts (For abbreviations see Materials and methods)		Apterous viviparae (n = 25)		
		Mean	Range	Standard deviation
Length (mm)	Body length	0.710	0.624–0.778	0.038
	Body width	0.524	0.422–0.581	0.042
	Whole antenna	0.065	0.059–0.074	0.004
	URS	0.029	0.026–0.031	0.001
	Hind trochanter and femur	0.076	0.072–0.082	0.003
	Hind tibia	0.090	0.084–0.096	0.004
	2HT	0.030	0.026–0.033	0.002
	Cauda	0.016	0.012–0.017	0.002
	BW Cauda	0.026	0.022–0.030	0.003
	Ant.IIIBD	0.011	0.010–0.014	0.001
	Frontal setae	0.032	0.028–0.037	0.003
	Submarginal setae on Tergite I	0.037	0.032–0.041	0.002
	Spinal setae on Tergite VIII	0.042	0.034–0.048	0.005
Ratio (times)	Whole antenna / Body	0.09	0.08–0.11	0.007
	Hind tibia / Body	0.13	0.11–0.14	0.007
	URS / BW URS	1.14	1.04–1.26	0.071
	URS / 2HT	1.01	0.92–1.13	0.082
	Cauda / BW Cauda	0.61	0.50–0.70	0.063
	Frontal setae / Ant.IIIBD	2.93	2.33–3.75	0.380
	Submarginal setae on Tergite I / Ant.IIIBD	3.39	2.58–4.25	0.446
	Spinal setae on Tergite VIII / Ant.IIIBD	3.82	2.80–5.00	0.701

*COI sequencing*. COI barcode sequence was obtained for the new species with primers LepF and LepR (Foottit et al. 2008) and has been deposited in GenBank.

*Specimen depositories*. The holotype and some paratypes of the new species and all examined specimens of *Parathoracaphis manipurensis* and *Parathoracaphis setigera* are deposited in the National Zoological Museum of China, Institute of Zoology, Chinese Academy of Sciences, Beijing, China (NZMC). Four paratypes of the new species and the examined specimens of *Parathoracaphis cheni* are deposited in the Natural History Museum, London, UK (NHM).

## Taxonomy

### Parathoracaphis

Taxon classificationAnimaliaHemipteraAphididae

Takahashi, 1958

Parathoracaphis Takahashi, 1958: 13. Type species: Thoracaphis setigera Takahashi, 1932; by original designation.
Hoplothoracaphis Pramanick, Samanta & Raychaudhuri, 1983: 1. Type species: Hoplothoracaphis manipurensis Pramanick, Samanta & Raychaudhuri, 1983; by monotypy.
Parathoracaphis Takahashi: Ghosh and Raychaudhuri 1973: 486; Eastop and Hille Ris Lambers 1976: 336; Ghosh 1988: 194; Tao 1990: 71; Blackman and Eastop 1994: 799; Remaudière and Remaudière 1997: 188; Tao 1999: 22; Nieto Nafría et al. 2011: 305.

#### Generic diagnosis.

In apterae, body elongate oval, oval, or subcircular, aleyrodiform, and strongly sclerotized. Prosoma consisting of fused head, thorax, and abdominal segments I–VII, abdominal segment VIII free. Dorsum of prosoma reticulated, corrugated, convoluted, or with wax pores. Submarginal setae on dorsal prosoma distinctly spine-like, pointed or somewhat blunt at apices, sometimes arising from tuberculate bases. Dorsal spinal setae on prosoma minute, long and fine, or spine-like. Abdominal tergite VIII with 4 setae, similar to submarginal setae on dorsal prosoma. Eyes 3-faceted. Antennae concealed under head, 2–4-segmented. Legs short, concealed under body; tarsi small, unsegmented or 2-segmented; claws small, normal, or absent. Siphunculi absent. Cauda knobbed and constricted at base. Anal plate bilobed.

#### Distribution.

China, India, Japan, Malaysia, and Thailand.

#### Host plants.

Fagaceae (*Castanopsis*, *Cyclobalanopsis*, *Lithocarpus*, and *Quercus*) and Lauraceae (*Litsea*).

#### Comments.

Only apterous viviparous females are known. The life cycles of most species are unknown. *Parathoracaphis* is related to *Neohormaphis* Noordam, 1991 in sharing the consolidated head, thorax, and abdominal segments I–VII and spine-like submarginal setae on prosoma.

### Parathoracaphis cheni

Taxon classificationAnimaliaHemipteraAphididae

(Takahashi, 1936)

Thoracaphis cheni Takahashi, 1936: 21.
Parathoracaphis cheni : Eastop and Hille Ris Lambers 1976: 336; Blackman and Eastop 1994: 799; Remaudière and Remaudière 1997: 188; Tao 1999: 22.

#### Specimens examined.

Twelve apterous viviparous females, **CHINA**: Zhejiang (Huangyan), Jan 1934, on *Myrica rubra*, coll. F.G. Chen (NHM).

#### Distribution.

China (Zhejiang).

#### Host plant.

The host plant is recorded as *Myrica rubra* with a question mark in the original description (Takahashi 1936). We think it may be an erroneous record.

#### Biology.

Sitting tightly on the undersides of leaves of host plant (Takahashi 1936, Blackman and Eastop 1994). The life cycle is unknown.

### Parathoracaphis elongata

Taxon classificationAnimaliaHemipteraAphididae

(Takahashi, 1941)

Thoracaphis elongata Takahashi, 1941: 22.
Parathoracaphis elongata : Takahashi 1958: 14; Ghosh and Raychaudhuri 1973: 486; Eastop and Hille Ris Lambers 1976: 336; Blackman and Eastop 1994: 799; Remaudière and Remaudière 1997: 188.

#### Distribution.

Thailand.

#### Host plants.

*Quercus* sp. and other unspecified Fagaceae species.

#### Biology.

This species occurs in large numbers on the undersides of leaves of host plant (Takahashi 1941). The life cycle is unknown.

#### Comments.

Known only from the original description. Takahashi (1941) mentioned that the dorsal prosoma of this species has 5 pairs of spine-like spinal setae, tarsi are unsegmented, and claws are absent.

### Parathoracaphis gooti

Taxon classificationAnimaliaHemipteraAphididae

(Takahashi, 1950)

Thoracaphis gooti Takahashi, 1950: 605.
Parathoracaphis gooti : Eastop and Hille Ris Lambers 1976: 336; Blackman and Eastop 1994: 799; Remaudière and Remaudière 1997: 188.

#### Distribution.

Malaysia.

#### Host plant.

*Quercus* sp.

#### Biology.

Infesting the undersides of leaves of host plant (Takahashi 1950). The life cycle is unknown.

#### Comments.

Known only from the original description. The figure in Takahashi (1950) indicates that the dorsal prosoma of this species has 16 pairs of spine-like submarginal setae and 5 pairs of long and fine spinal setae, a pair of submarginal setae on head dorsum located near the front end, and 2 pairs between the eyes. The original description mentioned that the antennae of *Parathoracaphis gooti* are 3- or 4-segmented, the tarsi are distinctly 2-segmented, and the claws are slender.

### Parathoracaphis kayashimai

Taxon classificationAnimaliaHemipteraAphididae

(Takahashi, 1950)

Thoracaphis kayashimai Takahashi, 1950: 602.
Parathoracaphis kayashimai : Takahashi 1958: 14; Ghosh and Raychaudhuri 1973: 486; Eastop and Hille Ris Lambers 1976: 336; Blackman and Eastop 1994: 799; Remaudière and Remaudière 1997: 188.

#### Distribution.

Malaysia.

#### Host plant.

*Quercus* sp.

#### Biology.

Feeding on the undersides of leaves of host plant (Takahashi 1950). The life cycle is unknown.

#### Comments.

Takahashi (1950) mentioned that *Parathoracaphis kayashimai* was closely related to *Parathoracaphis setigera*, differing in ornamentation of dorsum and leg measurements. The key to Nipponaphidini species on *Quercus* and the figure of *Parathoracaphis kayashimai* in Blackman and Eastop (1994) indicate that submarginal setae on abdominal tergite VI of this species are much smaller than setae on other tergites.

### Parathoracaphis manipurensis

Taxon classificationAnimaliaHemipteraAphididae

(Pramanick, Samanta & Raychaudhuri, 1983)

Figs 1–3

Hoplothoracaphis manipurensis Pramanick, Samanta & Raychaudhuri, 1983: 2.
Parathoracaphis manipurensis : Ghosh 1988: 196; Remaudière and Remaudière 1997: 188.

#### Specimens examined.

One apterous viviparous female, **CHINA**: Yunnan (Chuxiong City, Mt. Zixi), 22 Oct 2010, No. 24888, on *Castanopsis* sp., coll. X.L. Huang (NZMC); 1 apterous viviparous female, **CHINA**: Yunnan (Chuxiong City, Mt. Zixi), 22 Oct 2010, No. 24894, on Fagaceae, coll. X.L. Huang (NZMC).

#### Distribution.

China (Yunnan), India.

#### Host plants.

*Castanopsis* sp. and *Litsea sebifera*.

#### Biology.

Forming large colonies on the undersides of leaves of host plant, attended by ants sometimes (Fig. 2). Apterous adults bear much filiform and flocculent wax (Fig. 3). The life cycle is unknown.

**Figures 1–3.**
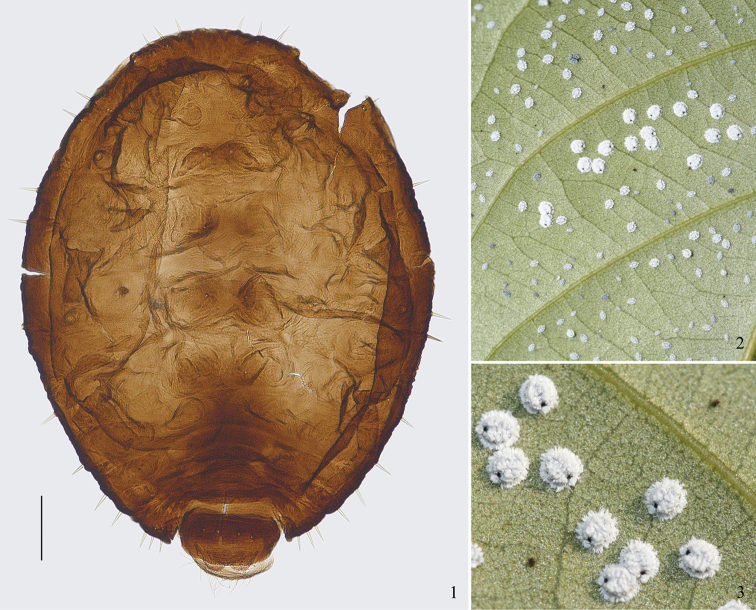
*Parathoracaphis manipurensis* (Pramanick, Samanta & Raychaudhuri). **1** Dorsal view of body of apterous viviparous female **2** a dense colony on underside of leaf of *Castanopsis* sp. **3** apterous adults in life, covered with much wax (**2, 3**: Mt. Zixi, Yunnan, China; 22 Oct 2010). Scale bar: 0.10 mm.

#### Comments.

Ghosh (1988) described an apterous morph on *Amaranthus* sp. (Amaranthaceae) as a fundatrix of *Parathoracaphis manipurensis*. However, the specimen was not collected at the type locality, no life cycle observations were conducted, and *Amaranthus* sp. is unlikely to be a primary host plant of Nipponaphidini species. Additionally, the presence of siphunculi suggests that it does not belong in *Parathoracaphis*.

### Parathoracaphis setigera

Taxon classificationAnimaliaHemipteraAphididae

(Takahashi, 1932)

Figs 4–7 , 11

Thoracaphis setigera Takahashi, 1932: 72.
Parathoracaphis setigera : Takahashi 1958: 14; Ghosh and Raychaudhuri 1973: 486; Eastop and Hille Ris Lambers 1976: 336; Tao 1990: 71; Blackman and Eastop 1994: 799; Remaudière and Remaudière 1997: 188; Tao 1999: 22.

#### Specimens examined.

Two apterous viviparous females, **CHINA**: Yunnan (Kunming City, 25.1407°N, 102.7465°E, altitude 1910 m), 18 Nov 2009, No. 23861, on *Quercus* sp., coll. J. Chen and Z.H. Luo (NZMC); 16 apterous viviparous females, **CHINA**: Yunnan (Kunming City, 25.0600°N, 102.7726°E, altitude 2000 m), 5 Dec 2009, No. 24111, on *Quercus* sp., coll. J. Chen and Z.H. Luo (NZMC); 17 apterous viviparous females, **CHINA**: Taiwan (Urai), 6 Sept 1931, No. Y7903, on *Quercus* sp., coll. R. Takahashi (NZMC).

#### Distribution.

China (Sichuan, Taiwan, and Yunnan), Japan.

#### Host plants.

*Cyclobalanopsis gilva*, *Lithocarpus* sp., *Quercus glauca*, and *Quercus myrsinaefolia*.

#### Biology.

Apterae are scattered on the undersides of leaves of host plant, with a circle of thin and curved wax filaments along the margin of body and two rather long wax filaments at the hind end of body (Fig. 7). In Japan, apterae occur on undersides of leaves of *Quercus* throughout the year (Takahashi 1958).

**Figures 4–7.**
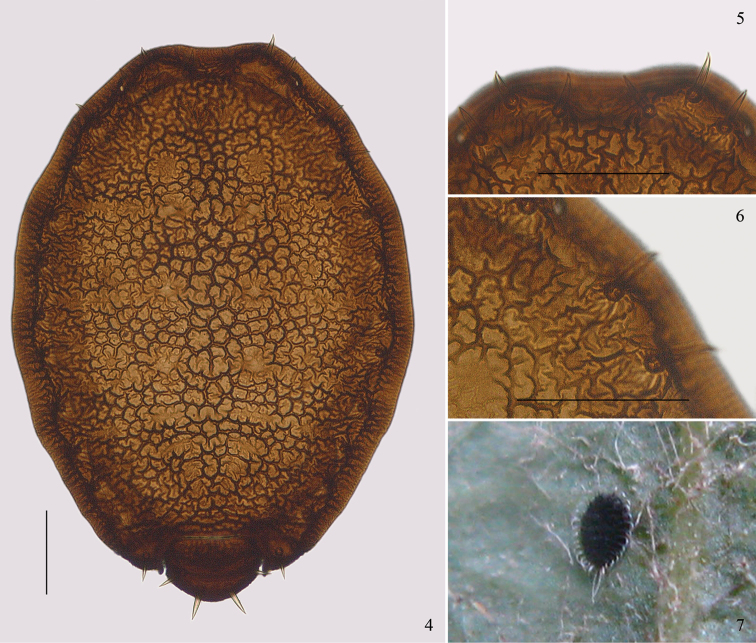
*Parathoracaphis setigera* (Takahashi). Apterous viviparous female: **4** dorsal view of body **5** submarginal setae on head dorsum **6** branched linear markings on marginal area of prosoma dorsum **7** apterous adult on underside of leaf of *Quercus* sp., bearing wax filaments marginally (Kunming, Yunnan, China; 5 Dec 2009). Scale bars: 0.10 mm.

### Parathoracaphis spinapilosa sp. n.

Taxon classificationAnimaliaHemipteraAphididae

http://zoobank.org/D5BEC449-00CA-4C14-83B6-5B3576BCC5E2

Figs 8–10 , 12–23 , 24–25 , Table 1

#### Etymology.

The new species is named for a pair of spine-like, long, thick, and pointed frontal setae. “*Spina*” (Latin) means “thorn”, “*pilosa*” (Latin) means “hair”.

#### Diagnosis.

Body small, aleyrodiform. Dorsum of prosoma densely covered with convoluted markings medially and short folded-line shaped sculptures pleuro-marginally. Head with a pair of spine-like frontal setae. Dorsum of prosoma with four pairs of minute spinal setae and 16 pairs of spine-like submarginal setae. Antennae 3- or 4-segmented. Tarsi 2-segmented. Claws normal.

#### Description.

*Apterous viviparous females*: Body oval, aleyrodiform, and strongly sclerotized (Fig. 12). Black in life, with a fringe of long and curved wax filaments, the filaments sparse at the hind end of body (Fig. 25). For morphometric data see Table 1.

#### Mounted specimens.

**Figures 8–11.**
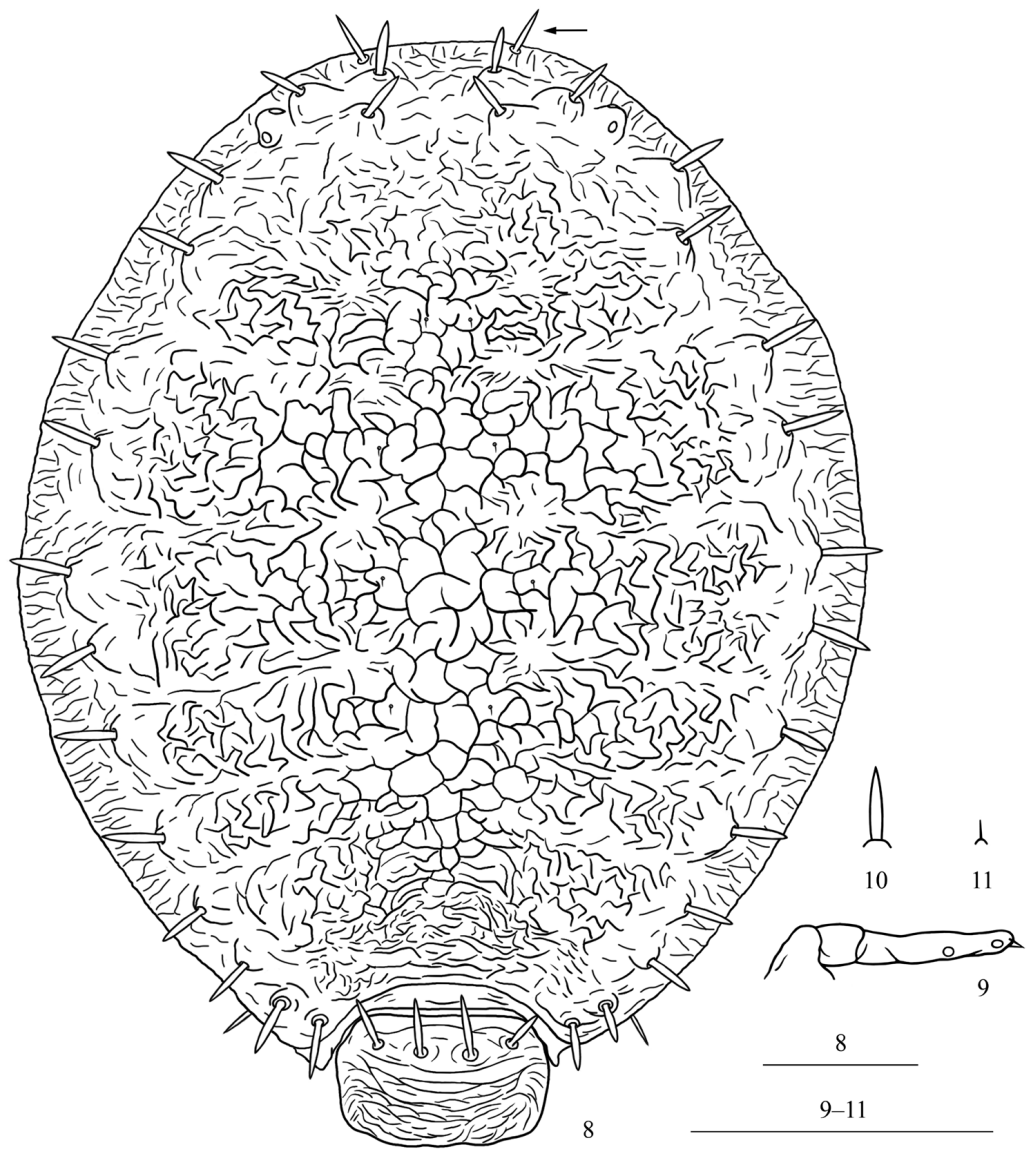
**8–9** *Parathoracaphis spinapilosa* sp. n. Apterous viviparous female: **8** dorsal view of body **9** antenna **10–11** Frontal seta: **10** *Parathoracaphis spinapilosa* sp. n. **11** *Parathoracaphis setigera*. Scale bars: 0.10 mm.

**Figures 12–23.**
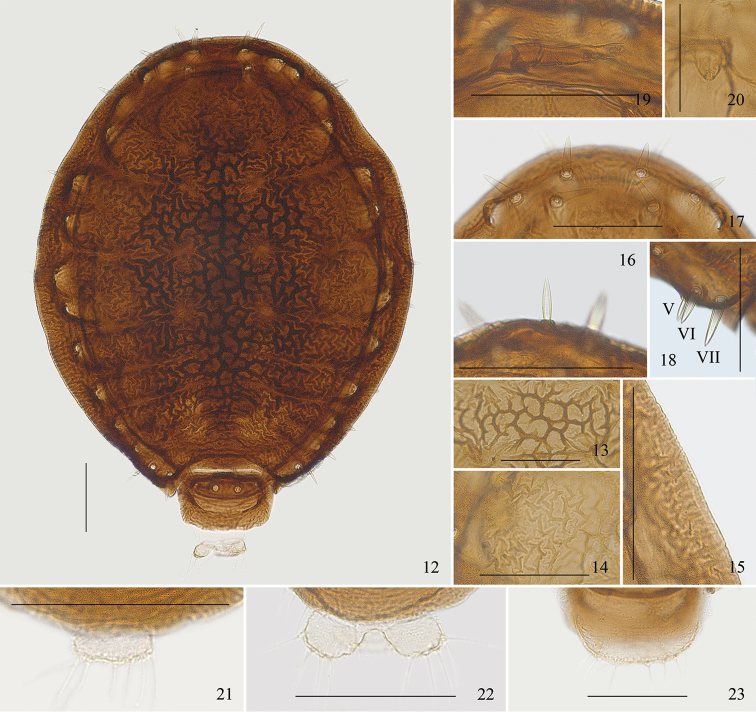
*Parathoracaphis spinapilosa* sp. n. Apterous viviparous female: **12** dorsal view of body **13** convoluted markings on medial area of prosoma dorsum **14** short folded-line shaped sculptures on pleuro-marginal area of prosoma dorsum **15** short transversely striped band on margin of prosoma **16** spine-like frontal seta on head **17** 3 pairs of submarginal setae on head dorsum **18** submarginal setae on abdominal tergites V–VII **19** antenna **20** ultimate rostral segment **21** cauda **22** anal plate **23** genital plate. Scale bars: 0.10 mm.

Body brown; cauda, anal plate, and genital plate pale in color. Prosoma consisting of fused head, thorax, and abdominal segments I–VII; abdominal segment VIII free (Figs 8, 12). Dorsum of prosoma densely covered with convoluted markings medially and short folded-line shaped sculptures pleuro-marginally (Figs 8, 12–14). The margin of dorsal prosoma with a short transversely striped band, band margin with small shallowly crenulated wax glands (Fig. 15). Between each segment of thoracic notum and abdominal tergites I–IV, shallow concave lines present at pleural and marginal area of prosoma; concave lines between abdominal tergites III and IV sometimes indistinct (Fig. 12). Abdominal tergite VIII with long and short ripples, distributed densely on posterior margin (Fig. 8). Head with a pair of frontal setae, spine-like, long, thick, and pointed (Figs 10, 16, indicated with an arrow in Fig. 8). Dorsum of prosoma with 16 pairs of long thick and spine-like submarginal setae, pointed or somewhat blunt at apices, arising from tuberculate bases (Fig. 8); head dorsum with two pairs anterior to eyes and a pair between eyes (Figs 8, 17), pro-, meso-, and meta-notum each with two pairs, abdominal tergites I–VII each with a pair (Fig. 8); submarginal setae on abdominal tergite V finer and shorter than setae on other tergites, pointed at apices, located near body margin (Fig. 18). Pro-, meso-, meta-notum, and abdominal tergite I each with a pair of minute spinal setae. Abdominal tergite VIII with four setae, similar to submarginal setae on dorsal prosoma (Fig. 8). Frons not protuberant. Eyes 3-faceted. Antennae 3-segmented, rarely 4-segmented, concealed under head, with two apical setae (Figs 9, 19). Primary rhinaria small, rounded, and placed wide apart. Rostrum short, reaching to fore coxae. Ultimate rostral segment short, thick, and blunt, with two pairs of primary setae and a pair of secondary setae (Fig. 20). Legs short, smooth, concealed under body, trochanter and femur fused. Tarsi 2-segmented. First tarsal chaetotaxy: 2, 2, 2. Dorsoapical setae on second tarsal segment expanded at apex and longer than claws. Claws normal. Siphunculi absent. Cauda and anal plate with spinules, genital plate with spinulose transverse stripes. Cauda knobbed, constricted at base, with six or seven setae (Fig. 21). Anal plate bilobed, each lobe with 4–6 setae (Fig. 22). Genital plate broadly rounded, with two anterior setae and 6–8 setae along the posterior margin (Fig. 23).

#### Specimens examined.

*Holotype*: apterous viviparous female, **CHINA**: Yunnan (Kunming City, Mt. Xishan), 24 Apr 1995, No. 13480-1-4-2, on Fagaceae, coll. G.X. Qiao (NZMC). *Paratypes*: 25 apterous viviparous females, with the same collection data as holotype (NZMC); 4 apterous viviparous females, No. 13480-1-5, with the same collection data as holotype (NHM); 9 apterous viviparous females (COI: KX709878), **CHINA**: Fujian (Jiangle County, Mt. Longqi, 26.5109°N, 117.2907°E, altitude 730 m), 17 Jun 2011, No. 26901, on *Quercus* sp., coll. J. Chen, Q.H. Liu, and X.T. Li (NZMC).

#### Taxonomic notes.

The new species resembles the type species *Parathoracaphis setigera* (Takahashi), but differs from it as follows: dorsum of prosoma densely covered with convoluted markings medially and short folded-line shaped sculptures pleuro-marginally (Figs 8, 12–14) (the latter with convoluted markings in medial and pleural area, and marginal area covered with branched linear markings radiating outwards, Figs 4, 6); head with a pair of long thick and spine-like frontal setae (Fig. 10) (in the latter: these are much shorter and finer, Fig. 11); dorsum of prosoma with 16 pairs of submarginal setae (the latter: 15 pairs, the pair on abdominal tergite V absent); head dorsum with two pairs of submarginal setae anterior to eyes, along the body margin, and a pair between eyes (Fig. 17) (the latter: all three pairs located along the body margin, Fig. 5); antennae 3- or 4-segmented (the latter: 2-segmented).

#### Distribution.

China (Fujian and Yunnan).

#### Host plants.

*Quercus* sp. and unidentified Fagaceae species.

#### Biology.

**Figures 24–25.**
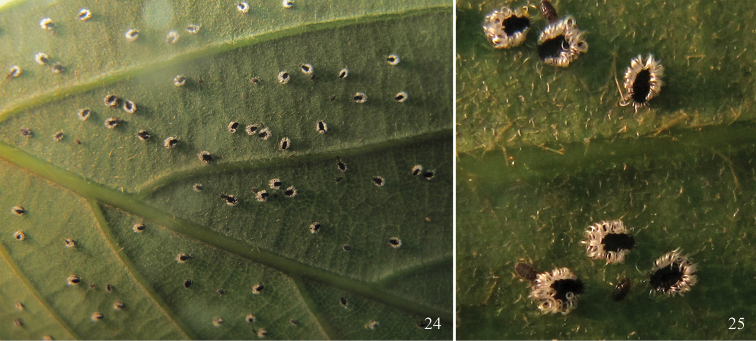
*Parathoracaphis spinapilosa* sp. n. (Mt. Longqi, Fujian, China; 17 Jun 2011) **24** A colony on underside of leaf of *Quercus* sp. **25** apterous adults in life, bearing long and curved wax filaments around the body.

Forming large colonies on the undersides of leaves of host plant (Fig. 24). The colony is attended by ants. Apterae bear long and curved wax filaments around the body (Fig. 25). The life cycle is unknown.

### Key to species of *Parathoracaphis* (apterous viviparous females)

**Table:** 

1	Dorsum of prosoma with 4 pairs of minute spinal setae (the pair on abdominal tergite II absent)	**2**
–	Dorsum of prosoma with 5 pairs of minute, long and fine, or spine-like spinal setae	**4**
2	Head with a pair of long thick and spine-like frontal setae; dorsum of prosoma with 16 pairs of spine-like submarginal setae; antennae 3- or 4-segmented	***Parathoracaphis spinapilosa* sp. n.**
–	Head with a pair of short, fine, and pointed frontal setae; dorsum of prosoma with 15 pairs of spine-like submarginal setae (the pair on abdominal tergite V absent); antennae 2-segmented	**3**
3	Submarginal setae on abdominal tergite VI much smaller than setae on other tergites	***Parathoracaphis kayashimai* (Takahashi)**
–	Submarginal setae on abdominal tergite VI thick spine-like, similarly sized with setae on other tergites	***Parathoracaphis setigera* (Takahashi)**
4	Dorsal spinal setae on prosoma spine-like, similar to submarginal setae; tarsi unsegmented, without claws	***Parathoracaphis elongata* (Takahashi)**
–	Dorsal spinal setae on prosoma minute or long and fine; tarsi 2-segmented, with claws	**5**
5	Dorsum of prosoma with 15 pairs of spine-like submarginal setae (the pair on abdominal tergite V absent); antennae 2-segmented	***Parathoracaphis cheni* (Takahashi)**
–	Dorsum of prosoma with 16 pairs of spine-like submarginal setae; antennae 3- or 4-segmented	**6**
6	Dorsum of prosoma without minute wax pores; head dorsum with a pair of submarginal setae near the front end and 2 pairs between eyes	***Parathoracaphis gooti* (Takahashi)**
–	Dorsum of prosoma densely covered with minute wax pores; head dorsum with 3 pairs of submarginal setae arranged in a row along the body margin	***Parathoracaphis manipurensis* (Pramanick, Samanta & Raychaudhuri)**

## Supplementary Material

XML Treatment for Parathoracaphis

XML Treatment for Parathoracaphis cheni

XML Treatment for Parathoracaphis elongata

XML Treatment for Parathoracaphis gooti

XML Treatment for Parathoracaphis kayashimai

XML Treatment for Parathoracaphis manipurensis

XML Treatment for Parathoracaphis setigera

XML Treatment for Parathoracaphis spinapilosa
